# Biometric Indices, Physio-Metabolic Responses and Carcass Quality in Rohu (*Labeo rohita*) during Feed Deprivation

**DOI:** 10.3390/ani12060769

**Published:** 2022-03-18

**Authors:** Sona Yengkokpam, Narottam Prasad Sahu, Asim Kumar Pal, Dipesh Debnath, Kamal Kant Jain, Rishikesh Subhashrao Dalvi, Petr Slama, Kavindra Kumar Kesari, Shubhadeep Roychoudhury

**Affiliations:** 1 Fish Nutrition, Biochemistry and Physiology Division, ICAR-Central Institute of Fisheries Education, Versova, Mumbai 400061, India; sonayen@gmail.com (S.Y.); npsahu1@rediffmail.com (N.P.S.); akpal53@gmail.com (A.K.P.); drkamaljain1955@rediffmail.com (K.K.J.); rishi.dalvi@gmail.com (R.S.D.); 2 Regional Centre, ICAR-Central Inland Fisheries Research Institute, Guwahati 781006, India; 3 Department of Zoology, Maharshi Dayanand College, Parel, Mumbai 400012, India; 4 Department of Animal Morphology, Physiology and Genetics, Faculty of AgriSciences, Mendel University in Brno, 61300 Brno, Czech Republic; petr.slama@mendelu.cz; 5 Department of Bioproducts and Biosystems, School of Chemical Engineering, Aalto University, 00076 Espoo, Finland; kavindra.kesari@aalto.fi; 6 Department of Life Science and Bioinformatics, Assam University, Silchar 788011, India

**Keywords:** aquaculture, Indian major carp, digestive enzymes, metabolic enzymes, antioxidative enzymes, starvation, condition factor, proximate composition

## Abstract

**Simple Summary:**

Feed deprivation is common among animals, including the aquatic animals. Because of this phenomenon, organisms may even suffer from severe metabolic disorders. In the present study, a rapid depletion in carbohydrate and lipid stores was noted in rohu fish (*Labeo rohita*), together with an elevation in gluconeogenic and antioxidant enzyme activities and inhibition of lipogenic enzyme activity under a diet-deprived condition. The biometric indices, physio-metabolic responses, and carcass quality in the rohu suggest that feed deprivation beyond 30 days caused an overall reduction in their metabolic activities. This is an interesting study, especially for aquatic habitats and aquatic life which may suffer from natural disasters, climate change impacts, or human activities altering their physio-metabolic functions.

**Abstract:**

Understanding changes in biometric indices and metabolism in fish exposed to feed deprivation may be useful in aquaculture. The present study elucidates the effect of feed deprivation on physio-biochemical responses, such as changes in biometric indices, nutrient mobilization patterns, and enzyme activities in rohu (*Labeo rohita*). Experimental fish (av. wt. 3.41 ± 0.07 g) were deprived of feed and sampled at intervals of 0, 15, 30, 45, and 60 days to measure weight, length, body composition, and the activities of enzymes involved in digestion, metabolism, and antioxidation. A decrease in body weight, condition factor (CF), hepatosomatic index (HSI), and gastrosomatic index (GSI) was observed during the initial stage of feed deprivation (15 to 30 days) but remained unchanged thereafter. The total carbohydrate and lipid content also decreased rapidly up until 30 days, then stabilized. However, the reduction in tissue protein content (% wet weight) continued gradually with the duration of feed deprivation from 12.85 ± 0.36 at 0 days to 10.04 ± 0.67 at 15 days, 8.79 ± 0.59 at 30 days, 6.95 ± 0.69 at 45 days, and 6.16 ± 0.8 at 60 days, which was lower, compared to the other two body constituents. Amylase, protease. and lipase activities significantly reduced up until 30 days, but then stabilized. Although G6PDH enzyme activity decreased, gluconeogenic (LDH, AST, and ALT) and antioxidative (SOD and catalase) enzyme activities increased during initial feed deprivation (up to 30 days). A tissue-specific difference in amino acid metabolism with a major role of ALT in liver and AST in muscle was observed. This study revealed that rohu fingerlings adapted well to feed deprivation up until 30 days, beyond which there was an overall deterioration in the metabolic functions.

## 1. Introduction

The feed deprivation of many fish species is considered as occurring in their natural environments due to seasonal changes, competition for food, and breeding migration [1]. Intentional feed deprivation episodes are also common in aquaculture practices, with the objective of achieving the advantage of compensatory growth [2,3]. The literature suggests that fish have the capacity to tolerate long periods of feed deprivation [4,5] through a series of adaptive physiological processes [1]. However, the nature of the response to this phenomenon, the types and/or patterns of nutrient reserves utilized, and the tissues from where these reserves are drawn differ from species to species [4]. In feed-deprived fish, metabolic demands are fulfilled by mobilizing stored body nutrients; even body constituents are mobilized in long-term feed deprivation to maintain life processes [6,7]. Carbohydrates, mostly in the form of glycogen, are stored in the liver and muscle of fish. Glycogen is the main substrate to be mobilized during the initial stage of feed deprivation [4,8,9]. However, its contribution to total energy production is comparatively small because glycogen is present in small amounts in fish (generally varying between 1–4%). Lipid is stored in the liver, intestine and muscles, and nutrient depletion from these tissues depends on the species, the type of tissue, and the utilization of other reserves, such as carbohydrates [1,4]. However, during prolonged feed deprivation, especially when lipid stores are depleted, muscle proteins are catabolized to produce amino acids as the major source of energy [10,11].

Few studies report the modulation of the enzymes of the digestive system and intermediary metabolism due to feed deprivation in fish. Studies in Atlantic salmon (*Salmo salar*) [12], Atlantic cod (*Gadus morhua*) [5], and Japanese flounder (*Paralichthys olivaceus*) [13] reported decreases in digestive enzyme activities following starvation. In contrast, an increase in metabolic enzyme activities was observed in the mucosa of the stomach and intestinal tract of Nile tilapia (*Oreochromis niloticus*) subjected to short-term fasting [14]. During starvation, the activities of the gluconeogenic enzymes are enhanced [15,16], while the activities of glucose-degrading enzymes and those of lipogenesis are inhibited [6,17].

For decades, Indian farmers have been practicing reduced/restricted feeding of fingerlings of Indian major carp (IMC) for a few months to obtain stunted fingerlings before stocking in grow-out ponds. The advantages of producing and stocking stunted fingerlings are direct savings on feed and labor costs and compensatory growth [2,3]. Rohu (an IMC) is an important freshwater species cultured on the Asian subcontinent. Although a few studies report the effects of short-term feed deprivation and refeeding in rohu [2,18,19], there is dearth of information on the changes in the biometric indices and the activities of the digestive and metabolic enzymes during long-term feed deprivation in this species. The present study was conducted on rohu with the aim of assessing changes in the biometric indices, nutrient mobilization patterns in tissues, and the activities of digestive, metabolic, and antioxidative enzymes in response to long-term starvation, which would help in furthering our understanding of the process of metabolic modulation to feed deprivation in aquaculture, rationally and scientifically.

## 2. Materials and Methods

### 2.1. Experimental Fish and Design

Rohu fingerlings (avg. wt. 3.41 ± 0.07 g) were procured from Palghar fish farm, Mumbai, Maharashtra, India, and transported to the wet laboratory of the Central Institute of Fisheries Education, Mumbai. Fish were acclimated to the laboratory conditions for one month and fed with a formulated diet containing 30% crude protein and 6% lipid until satiation, twice a day. A total of 120 fingerlings were equally distributed in 4 plastic tanks of 150 L capacity (i.e., 30 fish per tank) and allowed to acclimate to the experimental tanks for another 5 days, during which time they were fed with formulated feed. At the end of the 5-day acclimation period, feed was withheld, and the fish were randomly sampled at intervals of 0, 15, 30, 45, and 60 days of feed deprivation to measure body weight and length, and for biometric analysis, proximate analysis, and enzyme assays. Continuous aeration was provided in the tanks throughout the experimental period and 50% of the water was exchanged every alternate day to maintain water quality. The water quality parameters in the tanks were recorded by following the standard methods [20] and were found to be within the acceptable ranges for aquaculture (water temperature: 26.9–28 °C; dissolved oxygen: 6.7–7. 2 mg L^−1^; pH: 7.9–8.3; ammonia (NH_3_): 0.12–0.37 mg L^−1^; and nitrate 0.02–0.13 mg L^−1^).

### 2.2. Sampling

A total of 3 fish were collected randomly at the end of each sampling interval (0, 15, 30, 45, and 60 days), and euthanized with an overdose of clove oil (50 µL L^−1^ water). The liver, muscle, and whole intestine were excised by dissection for enzyme assays. Each tissue was then weighed and homogenized (5%; weight/volume) in a chilled sucrose solution (0.25 M) using a tissue homogenizer (MICCRA D-9, Digitronic, Bergheim, Germany). This was followed by the centrifugation of the homogenate at 10,000× *g* for 20 min at 4 °C, and the supernatant was collected in glass vials and stored at −20 °C until further analysis. Simultaneously, another set of 3 fish were sampled randomly from each tank for proximate composition analysis.

### 2.3. Biometric Analysis

Body weight and total length were measured for each euthanized fish (with a total of 6 fish from each tank). The biometric indices were calculated as follow:

Condition factor (CF) = (W/L^3^) × 100

Hepatosomatic index (HIS) = (Liver weight in g/W) × 100

Gastrosomatic index (GSI) = (Gastrointestinal tract weight in g/W) × 100

where, W is the weight of the fish in g, and L is the length of the fish in cm.

### 2.4. Proximate Composition Analysis

Analysis of the proximate composition of whole fish was performed using the standard methods [21]. The fish were dried to constant weight at 100 °C to determine the moisture content. Crude protein (CP) content was determined using a Kjeltec semi-automatic system (Foss Tecator, Hoeganaes, Sweden), and lipid content was assessed using a Soxtec system (Foss Tecator, Hoeganaes, Sweden), while ash content was determined by incineration in a muffle furnace at 550 °C for 6 h. The formula 100 − (moisture% + CP% + lipid% + ash%) was used to calculate the total carbohydrate content.

### 2.5. Assays of Digestive Enzymes

For the analysis of the digestive enzymes, the whole intestine was used. The amount of reducing sugar produced due to the action of glucoamylase and α-amylase on starch was measured to determine the amylase activity (E.C. 3.2.1.1) [22]. Starch solution (2%), phosphate buffer (0.1 M, pH 7), and tissue homogenate comprised the reaction mixture, which was incubated at 37 °C for 30 min. A coloring agent, dinitrosalicylic acid, was added to it, and the tubes were kept in boiling water for 5 min. The reaction mixture was diluted with distilled water after cooling, and absorbance was measured at 540 nm. The maltose standard curve was used to determine the amount of reducing sugar, and the activity of amylase was expressed as moles of maltose released from starch/min/mg protein at 37 °C.

The casein hydrolysis method was used to measure the total proteolytic activity [23]. The reaction mixture consisting of 1% (*w*/*v*) casein, phosphate buffer (0.1 M, pH 8.0), and tissue homogenate was incubated at 37 °C for 60 min. The addition of 6% trichloroacetic acid (TCA) was used to stop the reaction. The mixture was centrifuged at 10,000× *g* for 10 min after cooling at 4 °C, and the absorbance of the supernatant was recorded at 280 nm against a reagent blank. The tyrosine standard curve was used to determine the activity of the proteolytic enzyme, and it was expressed as moles of tyrosine released/min/mg protein at 37 °C.

Lipase (E.C. 3.1.1.3) activity was determined following the method of Cherry and Crandell [24]. Tissue homogenate, phosphate buffer (0.1 M, pH 7), and olive oil emulsion comprised the reaction mixture. The mixture was shaken well and incubated at 4 °C for 24 h. Then, 95% alcohol and 2 drops of phenolphthalein indicator were added and titrated against 0.05 N NaOH until the appearance of a permanent, pink color. Inactivated enzyme source prior to the addition of the buffer and olive oil emulsion comprised the reaction control. As a measure of the activity of lipase, the milliequivalent of alkali consumed was taken.

The spectrophotometric method of Garen and Levinthal was used to determine the alkaline phosphatase activity (ALP; E.C. 3.1.3.1) and acid phosphatase (ACP; E.C. 3.1.3.2) [25]. The reaction mixture comprised 0.2 M buffer (bicarbonate buffer, pH 9.4 for ALP or acetate buffer, pH 5.0 for ACP), 0.1 M MgCl_2_, 0.1 M paranitrophenyl phosphate, and tissue homogenate. The reaction was stopped by using 0.1 N NaOH, and absorbance was recorded at 410 nm. The activities of ALP and ACP were expressed as nanomoles p-nitrophenol released/min/mg protein at 37 °C.

### 2.6. Assays of Metabolic Enzymes

The muscle and liver tissues were used for the analysis of metabolic enzymes. All metabolic enzyme assays (except AST and ALT) were carried out at room temperature (30 °C). The method of Easterby and O’Brien was used to measure the hexokinase (HK; E.C. 2.7.1.1) activity [26]. The reaction cocktail was composed of Tris-HCl buffer (50 mM), glucose (50 mM), ATP (30 mM), and MgCl_2_ (200 mM). The pH was adjusted to 7.6 at 30 °C. The final reaction mixture consisted of reaction cocktail, β-NADP (1 mM), and G6PDH (500 U/mL). One unit of enzyme was defined as the amount of enzyme that phosphorylates 1.0 µmole of D-glucose/min at 30 °C.

The method of Wroblewski and Ladue was used to determine the activity of lactate dehydrogenase (LDH; E.C. 1.1.1.27) [27]. The reaction mixture comprised 0.1 M phosphate buffer (pH 7.5), NADH solution (0.2 mM), tissue homogenate, and sodium pyruvate (0.02 M), and the optical densities (OD) were recorded at 340 nm at 30 s intervals for 3 min. The reaction mixture for the estimation of malate dehydrogenase (MDH; E.C. 1.1.1.37) was similar to that of the LDH assay, except that 0.1 mM oxaloacetate was used as the substrate [28]. The method of De Moss was used to determine the activity of glucose-6-phosphate dehydrogenase (G6PDH; E.C. 1.1.1.49) [29]. The reaction mixture consisted of Tris-buffer (0.1 M, pH 7.8), freshly prepared NADH (2.7 mM), MgCl_2_ (0.1 M), and glucose-6-phosphate (0.2 M). Tissue extract or substrate was added to the reaction mixtures to initiate the enzymatic reactions, and subsequent changes in the absorbance of NADH or NADP were recorded at 340 nm to determine the enzyme activity. The activity of the LDH, MDH, and G6PDH enzymes was expressed as units/min/mg protein at 30 °C, where 1 unit corresponded to ∆0.01 OD/min.

Alanine amino-transferase (ALT; E.C. 2.6.1.2.) and aspartate amino-transferase (AST; E.C. 2.6.1.1.) were assayed following the method of Wootton [30]. For ALT estimation, the reaction mixture contained 200 mM DL-alanine and 2 mM α-ketoglutarate in 40 mM phosphate buffer (pH 7.4), and the OD was read at 540 nm. A similar assay mixture was used for AST, except that 200 mM DL-aspartic acid was used as the substrate instead of alanine. The enzyme activities were expressed as nanomoles oxaloacetate formed/min/mg protein at 30 °C.

### 2.7. Assays of Antioxidative Enzymes

The liver tissues were used for the analysis of the antioxidative enzymes. Catalase (E.C. 1.11.1.6.) activity was measured as the decrease in hydrogen peroxide (H_2_O_2_) concentration at 240 nm [31]. The reaction mixture consisted of phosphate buffer (50 mM, pH 7.0), tissue homogenate, and freshly prepared H_2_O_2_ (0.3%). The enzyme activity was expressed as nanomoles of H_2_O_2_ decomposed/min/mg protein.

Superoxide dismutase (SOD; E.C. 1.15.1.1.) activity was determined following the method of Misra and Fridovich [32]. The assay was based on the oxidation of epinephrine-adrenochrome transition by SOD. The reaction mixture consisting of carbonate-bicarbonate buffer (0.1 M, pH 10.2), tissue homogenate, and freshly prepared epinephrine (3 mM) was mixed well, and the change in absorbance was immediately read at 480 nm. One unit of SOD activity corresponded to the amount of protein required for 50% inhibition of epinephrine autooxidation.

Total protein content in all the tissue homogenates was determined following the method of Lowry et al. [33] using bovine serum albumin as a standard. All the colorimetric assays were carried out using a UV–Vis spectrophotometer (E-Merck, Darmstadt, Germany).

### 2.8. Statistical Analysis

The effect of duration of feed deprivation on the proximate composition, biometric parameters, and enzyme activities was analyzed by one-way analysis of variance (ANOVA), followed by Duncan’s multiple range test for post-hoc comparison of means using SPSS (Version 16.0). The homogeneity of variance and normality of data were verified before the application of the statistical tests. Data presented in the text, figures, and tables are expressed as mean ± standard error (SE), and the significance for all statistical comparisons was set at *p* < 0.05.

## 3. Results

### 3.1. Biometry

**Figure 1:**
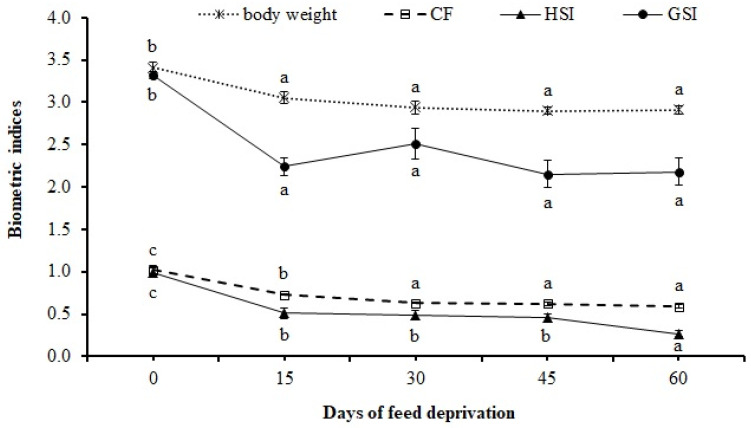
Body weight and biometric indices (CF, HSI, and GSI) of *L. rohita* fingerlings at 0, 15, 30, 45, and 60 days of feed deprivation. Data expressed as mean ± S.E. (*n* = 4). CF: Condition factor = [weight of fish/(length in cm)^3^] × 100; HSI: Hepatosomatic index = (weight of liver/weight of whole fish) × 100; GSI: Gastrosomatic index = (weight of intestinal tract/weight of whole fish) × 100. Different letters (a, b, c) on the same line signify statistical differences (*p* < 0.05) among the groups.

Changes in the biometric indices during feed deprivation in rohu fingerlings are depicted in Figure 1. The body weight and GSI were reduced significantly (*p* = 0.001) after 15 days of feed deprivation and remained unchanged thereafter. The HSI decreased significantly (*p* = 0.001) at 15 days, remained low up to 45 days, and later decreased significantly (*p* = 0.021) at 60 days. The CF reduced significantly (*p* < 0.001) up to 30 days with no further reduction.

### 3.2. Proximate Composition

The proximate composition of rohu fingerlings at different intervals of feed deprivation is presented in Table 1. The moisture content showed a gradual and significant (*p* = 0.01) increase with the increasing duration of feed deprivation, with a 9.6% increase until 60 days, compared to 0 days. The protein contents decreased significantly (*p* = 0.002) from 30 days until 60 days from those observed at 0 and 15 days. The total lipid and total carbohydrate contents gradually reduced (*p* = 0.001) up until 30 days and stabilized thereafter. However, the ash content showed a significant (*p* = 0.001) increase up until 30 days, after which the values stabilized.

**Figure 2:**
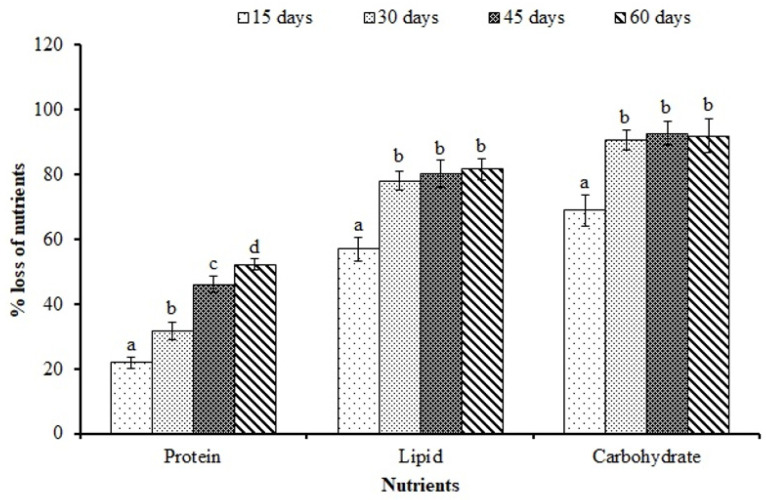
Percentage loss of nutrients (protein, lipids, and carbohydrates) in *L. rohita* fingerlings at 15, 30, 45, and 60 days of feed deprivation compared to those at 0 days. Data expressed as mean ± SE (*n* = 4). Different letters (a, b, c, d) over the bars of a nutrient signify statistical differences (*p* < 0.05).

Percentage losses in proteins, lipids, and carbohydrates at different durations of feed deprivation compared to those at 0 days are shown in Figure 2. The protein (%) decreased significantly (*p* < 0.05) with the increasing duration of feed deprivation and reached the maximum at 60 days. On the contrary, lipid and carbohydrate values significantly (*p* < 0.05) declined up to 30 days and then stabilized.

### 3.3. Digestive Enzymes

**Figure 3:**
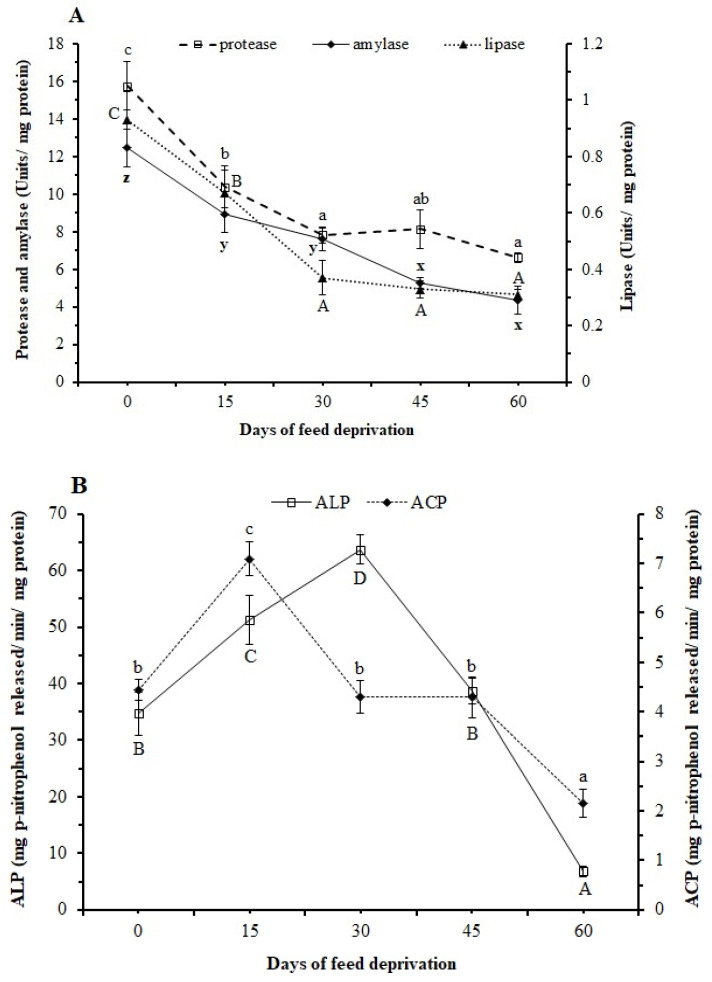
Activities of enzymes: (**A**) protease, amylase, and lipase; and (**B**) acid phosphatase (ACP) and alkaline phosphatase (ALP) in the intestine of *L. rohita* fingerlings at 0, 15, 30, 45, and 60 days of feed deprivation. Data are expressed as mean ± SE (*n* = 4). Different letters (a, b, c/A, B, C, D/x, y, z) on the same line signify statistical differences (*p* < 0.05).

Protease and lipase activities significantly (*p* < 0.05) declined up to 30 days, and later the activities remained unchanged (Figure 3A). Amylase activity declined significantly (*p* < 0.05) until 15 days and then remained unchanged at 30 days; it then declined again at 45 days (Figure 3A). However, the activities of intestinal ACP and ALP increased at 15 and 30 days, respectively, but later the values steeply declined, with a significant reduction at 60 days (Figure 3B).

### 3.4. Metabolic Enzymes

**Figure 4:**
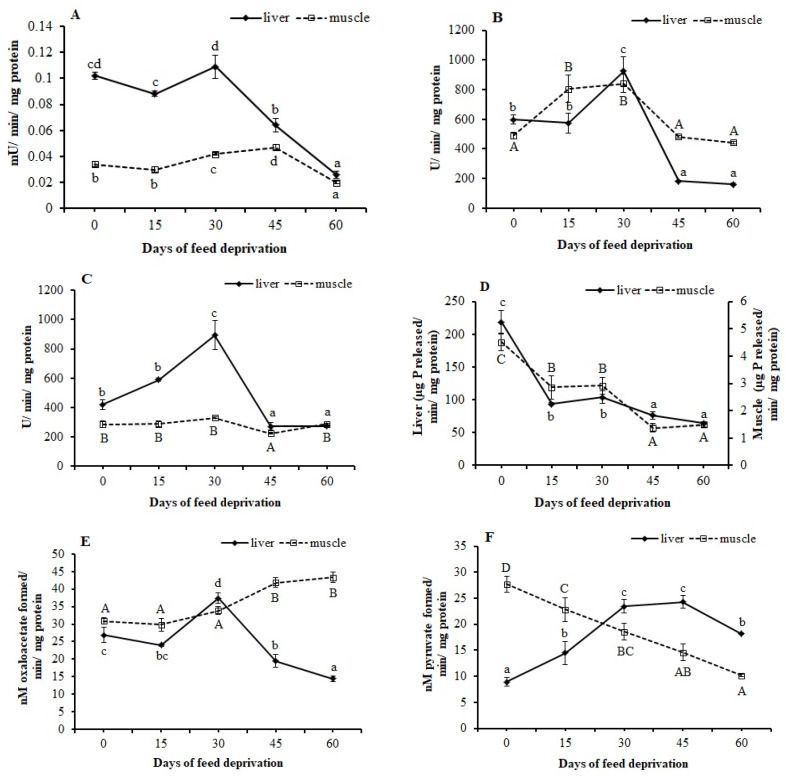
Metabolic enzyme activities in the liver and muscle of *L. rohita* fingerlings at 0, 15, 30, 45, and 60 days of feed deprivation: (**A**) Hexokinase; (**B**) LDH = lactate dehydrogenase; (**C**) MDH = malate dehydrogenase; (**D**) G6PDH = glucose-6-phospate dehydrogenase; (**E**) AST = aspartate amino-transferase; (**F**) ALT = alanine amino-transferase. Data expressed as mean ± SE (*n* = 4). Different letters (a, b, c, d; or A, B, C, D) on the same line signify statistical differences (*p* < 0.05).

Hexokinase, LDH, and MDH (except in muscle tissues) activities showed an increasing trend during the initial days of feed deprivation (Figure 4). The liver hexokinase activity slightly reduced at 15 days, later increased significantly (*p* < 0.05) at 30 days, and then decreased until day 60 (Figure 4A). The muscle hexokinase activity was stable until 15 days, later significantly (*p* < 0.05) increased at 30 days, and then reduced significantly (*p* < 0.01) at 60 days (Figure 4A). The liver LDH activity increased significantly (*p* < 0.01) at 30 days, and later decreased significantly (*p* < 0.05) at 45 days and 60 days (Figure 4B). The muscle LDH activity significantly (*p* < 0.05) increased at 15 days and later decreased at 45 and 60 days (Figure 4B). The liver MDH showed a trend similar to that observed for liver LDH activity (Figure 4C). However, the muscle MDH showed a significant (*p* < 0.05) decrease only at 45 days (Figure 4C).

The G6PDH activities showed a decreasing trend both in liver and muscle tissues (Figure 4D). The activities in both tissues initially decreased significantly (*p* < 0.01) at 15 days, remained stable up to 30 days, and further decreased significantly (*p* < 0.01) at 45 and 60 days.

The liver and muscle tissues showed an opposite trend in the AST and ALT activities (Figure 4E,F). Liver AST increased significantly (*p* < 0.01) at 30 days and later decreased (*p* < 0.05) at 45 and 60 days (Figure 4E). However, the muscle AST activity remained unchanged up to 30 days but increased significantly (*p* < 0.05) at 45 days, and later remained unchanged until 60 days (Figure 4E). The liver ALT significantly (*p* < 0.05) increased until 30 days, remained unchanged until 45 days, and later declined again at 60 days (Figure 4F). However, the muscle ALT activity showed a decreasing trend (*p* < 0.01) with increasing duration of feed deprivation (Figure 4F).

### 3.5. Antioxidative Enzymes

**Figure 5:**
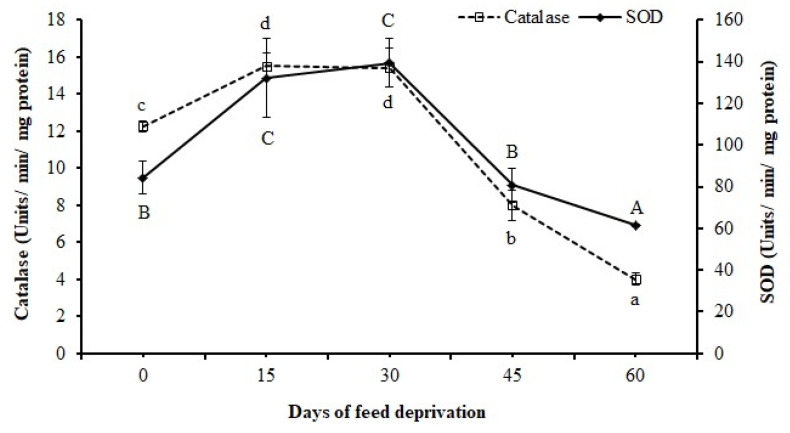
Superoxide dismutase (SOD) and catalase activities in the liver of *L. rohita* fingerlings 0, 15, 30, 45, and 60 days of feed deprivation. Data expressed as mean ± SE (*n* = 4). Different letters (a, b, c, d; or A, B, C) on the same line signify statistical differences (*p* < 0.05).

Both catalase and SOD activities showed significant (*p* < 0.01) increase at 15 days, remained unchanged up to 30 days, and later decreased significantly (*p* < 0.05) at 45 and 60 days of feed deprivation (Figure 5).

## 4. Discussion

Our results suggest that rohu fingerlings respond to feed deprivation by mobilizing the reserve carbohydrate followed by lipid reserves while conserving body protein stores to some extent. During the initial stage of feed deprivation (up to 15 days), a sharp reduction in body weight, GSI, and HSI indicated that feed deprivation significantly affected viscera and liver weight. This may be due to the preferential withdrawal of nutrient reserves (especially lipids) from the viscera and liver for maintenance energy during the first 15 days. A 10-week starvation study in white sturgeon (*Acipenser transmontanus*) showed a greater loss in the weight of the viscera and liver than the loss in the carcass weight [34]. Earlier reports on fasting (1–2 months) also reported little effect on body mass and length of fish [5,35,36]. One of the reasons for maintaining constant body weight after an initial loss is the replacement of catabolized lipids by an equal volume of water [11,37]. This is in agreement with our observation that the moisture content in rohu fingerlings gradually increased with the increasing duration of feed deprivation (Table 1).

Among the energy sources, greater reduction in lipid and carbohydrate contents observed until 30 days of feed deprivation suggested that these reserves were primarily mobilized during the initial stage of feed deprivation. We observed the maximum mobilization of carbohydrate with 68.9 and 90.5% reduction at 15 and 30 days of feed deprivation, respectively. This was followed by lipid stores, with 56.9 and 78% reductions at 15 and 30 days, respectively. However, the overall contribution of carbohydrates (mainly glycogen) towards total energy production is comparatively small considering its limited stores in the tissues, which amounts to 1–6% in the liver and 0.2–1.3% in the muscle [4]. Our results suggest that lipid is the major fuel for maintaining metabolic homeostasis until 15 to 30 days of feed deprivation in rohu. Hung et al. reported that, during the first four weeks of starvation in white sturgeon, there was a significant decrease in the carcass and visceral lipid contents, with conservation of membrane-associated phospholipids for maintaining cellular integrity [37]. Body lipids served as the major source of energy during periods of starvation, and a marked reduction in lipids occurred in starved fish [38,39,40]. In the present study, the extent of the reduction in tissue protein was lesser but gradual through the period of feed deprivation, with 21.9 and 52.1% decreases at 15 and 60 days, respectively, compared to that at 0 days, suggesting the conservation of body protein at the expense of stored lipids during feed deprivation. Our results are in accordance with those reported earlier in white sturgeon after 10 weeks of starvation with lower protein loss (9%) than lipid loss (84%) [34] and in rainbow trout (*Oncorhynchus mykiss*) (60% protein and 93% lipid loss) after 12 weeks of starvation [41]. The use of protein during starvation may not be strictly for energy production only, but it is possible that some protein could be converted to carbohydrate/sugar through gluconeogenesis and used as an energy source, particularly in the brain tissue [42], hence the conserved mobilization of the tissue protein. Earlier reports in juvenile tilapia (*Oreochromis niloticus* × *O. aureus*) [43] and barramundi (*Lates calcarifer*) [44] showed no significant change in ash contents after 3 weeks of starvation.

Immediate changes in digestive enzyme activities due to feed deprivation are attributed to the highly sensitive nature of the digestive organs in fish [12,13,45]. We observed a 29% reduction in intestinal amylase activity in the first 15 days, which reached 65% after 60 days of feed deprivation. The intestinal lipase activity declined significantly (*p* < 0.05) at 15 (29%) and 30 (60.2%) days and gradually declined to 66.7% at 60 days. Compared to the reduction in amylase and lipase activities, the reduction in protease activity was higher (34.2%) at 15 days, but the total reduction at 60 days (58%) was comparatively lower. These digestive enzymes act on the available substrates in the digestive tract (in the form of large protein, lipid, and carbohydrate molecules in feed) to break them down into smaller molecules for absorption. In the absence of feed, therefore, the activities of these enzymes are expected to decrease, as was observed in our study. Krogdahl and Bakke-McKellep reported in Atlantic salmon a rapid decrease in maltase and leucine aminopeptidase activities (up to 40%) during the first two days of starvation, which further continued to decline at a slower rate throughout the fasting period [45]. However, some amounts of digestive enzymes will be present in the intestine as these will be useful in digesting intestinal tissue lipids and proteins during feed deprivation episodes. Earlier studies in well-nourished fish suggested higher protein degradation in intestinal tissue than other tissues during the initial days of fasting [46,47]. As fasting continues, the catabolism of protein in the intestine eventually declines, with a concomitant shift to other tissues especially white muscle, providing amino acids for vital body functions [4]. We did not measure the total protease activity in the muscle; however, a gradual decrease in the whole-body crude protein content up to 60 days may be due to the gradual but slow breakdown of intestinal, as well as muscle, tissue proteins.

Intestinal alkaline phosphatase and acid phosphatase catalyze the hydrolysis of various phosphate-containing compounds and act as transphosphorylases at alkaline and acid pH, respectively. Alkaline phosphatase is considered a general marker of nutrient absorption [48], which is localized in the microvilli of the intestinal epithelium. Acid phosphatase is present mainly in the lysosomes and is related to the intracellular digestive enzyme and the maturation of the intestinal epithelium [49]. In our study, increased ALP activity up until 30 days and ACP up until 15 days indicated an enhanced efficiency of these enzymes for efficient nutrient absorption from the intestinal lumen and epithelium during the initial stage of starvation. Alternatively, as the external source of nutrient supply was cut off, the activities of these enzymes may have increased to salvage available nutrients. Earlier reports suggest that the breakdown of endogenous proteins leads to the formation of small peptides and amino acids, which are transported through the intestinal membrane, involving the collaboration of ALP and ATPase enzymes [50].

Hexokinase, a key glycolytic enzyme, is generally inhibited in fish upon starvation [51,52]. In the present study, noninhibition of hexokinase activity during the first 15 days of feed deprivation indicated that the energy demand of the fish was fulfilled through glycolysis and/or other mechanisms. With further increase in the duration of feed deprivation (until 30 days), glucose supply seemed to have come from the enhanced activity of the hexokinase enzyme, which may be corroborated by the severe loss of tissue carbohydrate content (90.5%) at 30 days of feed deprivation. Minimum values of liver and muscle hexokinase activity at 60 days may suggest the minimum availability of its substrate. The liver MDH activity (the representative enzyme for the citric acid cycle) was found to increase significantly at 30 days of feed deprivation, presumably due to an increased supply of acetyl CoA (the first substrate of the citric acid cycle) from lipolysis. This may be supported by the significantly reduced body lipids at 30 days of feed deprivation. Significantly reduced MDH activity corresponding to the stable body lipid content at 45 days of feed deprivation indicates decreased lipolytic activity.

G6PDH is one of the cytosolic enzymes supplying reducing equivalents in the form of NADPH and is considered ‘lipogenic’. Starvation causes the activation of lipolytic machinery and inhibits lipogenic activity [4]. We observed decreased G6PDH activity in both liver and muscle, indicating limited lipogenic activity during feed deprivation, similar to those reported earlier [34,52].

A variety of substrates including lactate, amino acids, and glycerol are used for gluconeogenesis by both intact liver and isolated hepatocytes [53]. In general, lactate gluconeogenesis is favored over alanine [15]. In the present study, the enhanced activity of the LDH enzyme in both liver and muscle tissues until 30 days of feed deprivation indicates increased gluconeogenesis (using lactate as the substrate). However, beyond 30 days of feed deprivation, the reduced LDH activities indicate the reduction in substrate supply and overall metabolic rate. Increased AST and ALT activities indicate increased gluconeogenic flux from amino acids during starvation in fish. Alanine and glutamate are excellent gluconeogenic substrates in most fish during starvation [15,54]. We observed a tissue-specific difference in metabolic adjustments via the amino acid catabolizing enzymes. In the liver, the increase in ALT activities observed until 45 days of feed deprivation indicates alanine as an important substrate for gluconeogenesis.

In the present study, the increased activities of the antioxidative enzymes, namely SOD and catalase, during the initial stage of feed deprivation suggests increased production of reactive oxygen species (ROS) and its neutralization. SOD reduces the ROS into H_2_O_2_, while catalase scavenges H_2_O_2_; thus, these enzymes protect the cell against oxidative damage [55]. However, beyond 30 days of feed deprivation, the decreased antioxidative enzyme activities may be attributed to the lesser production of ROS due to the reduced metabolic activities in rohu (as evidenced by the reduced metabolic enzyme activities that are mostly oxidative in nature). An increase in the activities of anti-oxidative enzymes was reported in fish during starvation as a defense against oxidative damage [6,55,56].

## 5. Conclusions

The response of rohu fingerlings to feed deprivation can be summarized in two stages. In the initial stage (until 30 days), there was a decrease in body weight, biometric indices, and nutrients, while in the later stage (beyond 30 days) there was no significant decrease in those parameters. While the activities of enzymes involved in glycolysis, the citric acid cycle, gluconeogenesis, and antioxidation were maintained or even enhanced during the initial stage of feed deprivation, the activities of digestive and lipogenic enzymes were inhibited. Feed deprivation beyond 30 days caused overall reduction in metabolic activities. The present study has elucidated various physio-metabolic changes in response to feed deprivation in fish, which might help us to set limits for feed restriction in aquaculture practices without affecting the health of the fish.

## Figures and Tables

**Table 1:** Proximate composition of *L. rohita* fingerlings (% wet weight) at 0, 15, 30, 45, and 60 days of feed deprivation.

Days	Moisture	Protein	Lipid	Total Carbohydrate	Ash
0	78.78 ^a^ ± 1.88	12.85 ^d^ ± 0.36	4.18 ^c^ ± 0.37	1.48 ^c^ ± 0.10	2.71 ^a^ ± 0.26
15	82.76 ^b^ ± 0.83	10.04 ^cd^ ± 0.67	1.83 ^b^ ± 0.21	0.46 ^b^ ± 0.13	4.91 ^b^ ± 0.02
30	83.46 ^bc^ ± 2.81	8.79 ^c^ ± 0.59	0.92 ^a^ ± 0.13	0.14 ^a^ ± 0.02	6.70 ^c^ ± 0.11
45	85.13 ^bc^ ± 1.27	6.95 ^b^ ± 0.69	0.83 ^a^ ± 0.12	0.11 ^a^ ± 0.05	6.98 ^c^ ± 0.51
60	86.39 ^c^ ± 1.32	6.16 ^a^ ± 0.8	0.77 ^a^ ± 0.03	0.12 ^a^ ± 0.02	6.56 ^c^ ± 0.12
*p* value	0.01	0.002	0.001	0.001	0.001

Values are expressed as the percentage of body weight. Values in the same column with different superscripts (a, b, c, d) differ significantly (*p* < 0.05) among the treatment groups. Data expressed as mean ± SE (*n* = 4).

## Data Availability

Not applicable.
